# Precision medicine for hepatocellular carcinoma: driver mutations and targeted therapy

**DOI:** 10.18632/oncotarget.18382

**Published:** 2017-06-06

**Authors:** Xiao-Xiao Ding, Qing-Ge Zhu, Shi-Ming Zhang, Lei Guan, Ting Li, Lei Zhang, Shi-Yang Wang, Wan-Li Ren, Xue-Mei Chen, Jing Zhao, Song Lin, Zhi-Zhen Liu, Yan-Xia Bai, Bing He, Hu-Qin Zhang

**Affiliations:** ^1^ The Key Laboratory of Biomedical Information Engineering of Ministry of Education, School of Life Science and Technology, Xi’an Jiaotong University, Xi’an 710049, P. R. China; ^2^ Department of Otorhinolaryngology - Head and Neck Surgery, The First Affiliated Hospital, Jiaotong University, Shaanxi 710061, P. R. China

**Keywords:** hepatocellular carcinoma, driver mutations, driver identification, targeted therapy, precision medicine

## Abstract

Hepatocellular carcinoma (HCC) is the third most frequent cause of tumor-related mortality and there are an estimated approximately 850,000 new cases annually. Most HCC patients are diagnosed at middle or advanced stage, losing the opportunity of surgery. The development of HCC is promoted by accumulated diverse genetic mutations, which confer selective growth advantages to tumor cells and are called “driver mutations”. The discovery of driver mutations provides a novel precision medicine strategy for late stage HCC, called targeted therapy. In this review, we summarized currently discovered driver mutations and corresponding signaling pathways, made an overview of identification methods of driver mutations and genes, and classified targeted drugs for HCC. The knowledge of mutational landscape deepen our understanding of carcinogenesis and promise future precision medicine for HCC patients.

## INTRODUCTION

Hepatocellular carcinoma (HCC) is one of the most mortality malignant tumors and has an incidence of approximately 850,000 new cases per year. HCC is often considered to be linked to multiple risk factors [1, 2], such as hepatitis B (HBV, 54%) and C (HCV, 31%) viral infections [3], high intake of alcohol, obesity and hemochromatosis. In North America, Europe and Japan, HCV is the leading cause of HCC, while HCC is most frequently associated with chronic HBV infection in Africa and many Asian countries [4]. To date, the best approach to prevent HCC is preventing the underlying liver disease, the best of which is the hepatitis B vaccination [5]. A significant reduction in the incidence of hepatocellular carcinoma has been observed in children aged 6 to14 years following a nationwide hepatitis B vaccination in Taiwan [6]. And another study provided evidence that hepatitis B vaccination was also effective to prevent HCC in young adults [7].

The Barcelona Clinic Liver Cancer (BCLC) staging system has been accepted worldwide in clinic practices and used in many clinic trials to developed new drugs for management of HCCs [8, 9]. HCC can be divided into five stages (0-D) in accordance with BCLC system and five corresponding treat methods are allocated: surgical resection, liver transplant, local ablation, transarterial chemoembolization (TACE) and sorafenib [10]. Only one-third of the HCC patients (stage 0-A) are applicable for curative therapies: surgical resection, liver transplant and local ablation [11]. There is a difficulty in diagnosis for early-stage HCCs due to atypical radiological appearance. In addition, most HCC patients are diagnosed at more advanced stages with only two managements showing growth advantages. Patients at stage B benefit from TACE method [10] with an estimated median survival of 26 months [12, 13]. The unfortunate reality is that patients at advanced HCC (stage C) only benefit from systematic therapy sorafenib with an increased median overall survival from 7.9 to 10.7 months before 2017 [14]. However, sorafenib treatment often induces some adverse events, such as hypophosphatemia, diarrhea and loss of weight. Thus, it is urgent to develop novel therapeutic strategies and drugs for HCC patients.

Like other solid tumors, HCC is caused by an accumulation of a series of gene mutations conferring a selective growth advantage to tumor cells, that sorts of mutations are called “driver mutations”. It has been estimated [15] that each driver mutation can provide a little selective growth advantage to the tumor cell, approximately 0.4% increase in difference between cell birth and cell death. Of course, a driver gene may contain some mutations having no effect on tumorigenesis, and such mutations are called “passenger mutations”. There has been an estimated that each HCC tumor possesses 30-40 mutations, among which 5-8 are considered drivers [11, 16]. The identification of driver mutations provides a potential therapy target for HCC patients.

In this review, three aspects will be included: an overview of driver mutations in HCC, the identification of drivers and targeted therapies for HCC.

## DRIVER MUTATIONS IN HCC

Plentiful studies revealed that each solid tumor is a unique and complex combination of series of somatic mutations driving the tumorigenesis. Like most solid tumors, there have been plentiful somatic mutations revealed relative to the development of HCC. By far, studies of mutational extents have concentrated on several genes, TERT, TP53, CTNNB1, ARID1A, ADRI2, NFE2L2 and KEAP1 included (Table 1). In addition, several major pathways are mostly aberrant in HCC, including telomere maintenance, TP53/cell cycle, WNT/β-catenin, chromatin remodeling, PI3K/RAS/mTOR pathway, oxidative stress pathways (KEAP1-NRF2 pathway) and angiogenesis. In this section, all above genes and corresponding pathways will be introduced, along with their impacts on the development of HCC.

**Table 1:** Genes most frequently mutated in hepatocellular carcinoma

Pathways	Genes	Function	Frequency in HCC (%)	References
Telomere maintenance	TERT	Maintaining telomere length	47.1	[20]
Cell cycle control	TP53	Tumor suppressor	28-36	[20, 29]
	CCND1	Cell proliferation	7.2	[11]
	CDKN2A	Cell cycle regulator	7.2	[3]
WNT-β-catenin signaling	CTNNB1	Transcriptional regulator	17-37	[20, 25, 33, 46]
	AXIN1	Signal transducer	4-14	[20, 29]
Oxidative stress	NFE2L2	Transcriptional regulator	6.4	[3]
	KEAP1	Proteinase adaptor	8	[46]
Chromatin remodeling	ARID1A	Chromatin remodeling	16.8	[3]
	ARID2	Chromatin remodeling	5.6	[3]
AKT-mTOR-MAPK pathway	RPS6KA3	kinase	2-10	[3, 29]
	PTEN	Tumor suppressor	3	[25]
	FGF19	Metabolic regulation factor	5	[25]
	PI3KCA	Effector of PTEN-AKT pathway	2-4	[3, 29]
JAK/STAT signaling	JAK1	kinase	5	[20]
Angiogenesis	VEGFA	Tumor proliferation	3.8	[54]

ARID: AT-rich interaction domain; AXIN1: axin 1; CCND1: cyclin D1; CDKN2A: cyclin-dependent kinase inhibitor 2A; CTNNB1: β-catenin; FGF19: fibroblast growth factor 19; KEAP1: kelch like ECH associated protein 1; NFE2L2: nuclear factor, erythroid 2 like 2; PI3K: phosphoinositide 3-kinase; PTEN: phosphatase and tensin homologue; RPS6KA3: ribosomal protein S6 kinase 90kDa, polypeptide 3; TERT: telomerase reverse transcriptase; TP53: tumor protein p53; VEGFA: vascular endothelial growth factor A.

### TERT, telomere maintenance

Telomeres are some short-repeated sequences (TTAGGG) located at terminal of each chromosome [17]. The telomerase complex is to maintain the length of each chromosome to avoid DNA damage and is composed of telomerase reverse transcriptase (TERT), which is the vital catalytic enzyme and plays an important role in cellular immortalization [18]. The activity of TERT is inhibited in human normal tissues while is reactivated in tumors. The TERT promoter region is composed of 260 base pairs with plentiful transcriptional factor binding sites, such as MYC and sp1. In particular, TERT promoter mutation is considered to be the most frequent alteration in HCC [19, 20]. The aberration of TERT has shown to be a prevalent property in human solid tumors with more than 95% of the tumors showing an upregulation of it [21]. And the unregulated mechanism may be attributed to the aberrant amplification of TERT genes or other deregulations [22].

The mutations in TERT promoter region provide some possible binding sites for E-twenty six/ternary complex factors (ETS/TCF) transcription factors and improve the activity of the promoter and transcriptional level [23, 24]. Interestingly, some investigators found that TERT mutations were significantly associated with CTNNB1 mutations in HCC, indicating that the interaction between TERT mutations and dysregulation of WNT-β-catenini pathways could promote the malignant transformation of HCC [19, 25, 26].

### TP53, cell cycle control

As a tumor suppressor gene, tumor protein 53(TP53) is always considered to be the second most frequent gene in HCC [27, 28], with a high mutation frequency of more than 30% [29]. And it encodes a functional transcription factor that responds to cellular stress, DNA replication and genomic stability [30]. In addition, TP53 also plays an important role in preventing cell abnormal proliferation. Somatic mutations of TP53 gene also are different in most tumors due to the heterogeneity of HCC samples [31]. There have been many studies to investigate the effects of TP53 mutations to signal pathway. And TP53 mutations are associated with poor prognosis in HCC, especially the hot mutations R249S and V157F. However, some mutations among the TP53 gene not only lost the function to depress the tumors’ development, but also can obtain a new contribution to promote liver cancers.

### CTNNB1 and AXIN1, WNT-β-catenin pathway

Catenin beta 1 (CTNNB1), encoding β-catenin, is one of the most frequent mutated genes, which can be observed in 20-30% cases in HCC [1, 32, 33]. Tumor progression and poor prognosis are often affected by the accumulation of β-catenin through WNT pathway. As illustrated below, when WNT signal is insufficient, β-catenin combines to the cytoplasmic compounds mediated by scaffold protein Axin and then is phosphorylated by the glycogen synthase kinase 3(GSK3). Once phosphorylated, recognition and degradation of β-catenin are followed due to the proteasome [34], which causes the low level of β-catenin and the inhibition of transcription for its targeted gene. In contrast, cytoplasmic compounds degrade when WNT ligands bind to the cell surface receptor (Frizzled, Fz) and co-receptor (LDL-receptor-related protein, LRP), which can effectively avoid the phosphorylation of β-catenin and maintain its stability [35]. And then β-catenin will be transferred to cell nucleus and make a combination with nuclear transcription factor TCF to regulate the expression of its targeted genes involved in cell growth. In addition, the aberrant activation of WNT signaling pathway plays a vital role in the occurrence and development of many human diseases, such as tumorigenesis, nervous system degenerative diseases.

AXIN1 is the second most frequently mutated gene in WNT pathway. And as a tumor suppressor gene, its function is to promote the phosphorylation of β-catenin and following degradation. In Taniguchi's study [36], the mutated frequency of AXIN1 was 9.6% in HCC.

### ARID1A and ARID2, chromatin remodeling

AT-rich interactive domain-containing protein 1A (ARID1A) is one of the key subunits of the SWI/SNF (switch/sucrose non-fermentable) complex remodeling chromosome [37, 38]. The SWI/SNF complex utilizes the energy from ATP to mobilize nucleosome to regulate the activity of DNA. As a tumor suppressor gene, its expression in normal liver tissues is abundant to restrain cell proliferation. Once mutated, its knockdown will have a promotive effect on development, migration and proliferation of HCC cells [39, 40]. To date, many investigators have found that mutational rate of ARID1A gene is approximately 10-15% in HCC. All these ARID1A mutations include missense, nonsense and frame shift mutations, leading to the down-regulation of its encoded protein.

AT-rich interactive domain 2 (ARID2) is another most frequent mutated genes in SWI/SNF complex. And as a tumor suppressor gene, frequency of inactivating mutations was discovered for 5-10% [41]. Mutations in ARID2 are lightly less than in ARID1A and can be divided into three types, including nonsense mutations, frame shifting indels and splice site mutations [42]. Duan et al. found that expression of ARID2 is significantly down-regulated in HCC compared with normal hepatic cells, which indicates that ARID2 is a tumor suppressor to inhibit cellular proliferation and growth of HCC cells [41]. Further researches suggested that ARID1A mutations were more common in alcohol intake-related HCCs than other risk factors, while ARID2 mutations frequently existed in HCV-related HCCs [42–45].

### NFE2L2 and KEAP1, oxidative stress pathway

Many studies have revealed that the oxidative stress pathway is significantly associated with the transformation and development of HCC [3, 25, 46]. Nuclear factor erythroid 2-related factor 2 (NFE2L2) plays a pivotal role in cellular anti-oxidation to prevent liver cells from a series of insults. Guichard and colleagues found that this pathway was activated in 12% of HCC samples with the two major mutations of NFE2L2 and Kelch-like ECH-associated protein 1 (KEAP1), with a frequency of 6.4% and 8%, respectively. And further study suggested that mutations in NFE2L2 and KEAP1 occurred in advanced HCC. In recent studies, there were some empirical evidence that the aberration in oxidative stress pathway could cooperate with WNT-β-catenin pathway to promote hepatocarcinogenesis [35].

### RPS6KA3, PTEN and FGF19, AKT-mTOR-MAPK pathway

As one of the most frequent mutated pathways, several genes are involved in HCCs. Some investigators found that RPS6KA3, a gene located at chromosome x that encodes ribosomal S6 protein kinase 2 (RSK2), was mutated in a frequency of 2-9% in HCC. RSK2 is the RAS inhibitor and the lasting activation RAS has been associated with HCC resistance to drugs, such as sorafenib. The phosphatase and tensin homologue (PTEN) mutations can be found in 1-3% HCC cases.

Fibroblast growth factor 19 (FGF19), transmitting signals through FGFR4 in mature liver cells, was mutated approximately 4-6% HCC patients and had an activation effect on PI3K-AKT-mTOR pathway [3, 47]. Recent studies found that inhibiting interaction between FGF19 and FGFR4 through an anti-FGF19 monoclonal antibody could effectively prevent HCC in transgenic mice and FGF19 provided a biomarker for HCC patients treated with anti-FGF19 therapy [11, 47, 48]. Sawey et al. proved that FGF19 plus CCND1, both located in chromosome regions 11q13, could increase mortality than either FGF19 or CCND1 group, suggesting the combination of the two genes were more effective to hepatocarcinogenesis.

Apart from FGFR, this pathway can also be activated by other tyrosine kinase receptor, including EGFR and insulin-like growth factor receptor (IGFR). Some studies found that EGF and EGFR were overexpression, which was associated with proliferation and differentiation of tumor cell [49]. And its overexpression shows a negative correlation with prognosis of HCCs, thus EGFR might be a potential target for HCC treatment.

Recent studies have found that mTOR pathway played a vital role in hepatocarcinogenesis [50]. mTOR pathway is more significantly altered in poor differentiation, vascular invasion, and higher stage tumors, and tumors with poor prognostic features [51]. Thus, patients with a highly activated mTOR pathway may benefit most from therapeutic strategy specifically targeting this pathway [52].

### VEGFA and PDGF, angiogenesis

Apart from DNA amplification in chromosome region 11q13, vascular endothelial growth factor A (VEGFA) situating in 6p21 is another genomic amplification in 3-7% HCC patients, which plays a pivotal role in promoting proliferation and migration of endothelial cells and improving vascular permeability [53, 54]. The mRNA and protein levels in HCC tumor cells are significantly higher than those in para-carcinoma tissue (P<0.01), and the overexpression of VEGFA could promote paracrine secretion of hepatocyte growth factor (HGF) [55, 56]. Moreover, that HGF binding to its specific receptor MET could promote epithelial cell migration. In addition, high levels of VEGF also are linked to poor overall survival (OS) and lower progression-free survival (PFS) in HCC patients. And further studies demonstrated that VEGFR inhibitors cabozantinib and ramucirumab showed an anti-tumor activity in HCC through interdicting VEGFR2 [57, 58], but results need to be validated in further clinical trials.

Platelet-derived growth factor (PDGF) and PDGFR, one of the angiogenic factors, have an intimate relativity with the development and progression of HCCs [59]. PDGF not only is indispensable in the proliferation and migration of vascular endothelial cells, smooth muscle cells and tumor cells, but also inhibits their apoptosis. Four PDGF ligands (PDGF-A,-B,-C,-D), bind to two different receptors, PDGFR α and PDGFR β. In many previous studies, PDGFR α is upregulated in HCC tissues when compared with adjacent liver tissues (p<0.01) and PDGF-A shows a consistent increase with PDGFRα expression [60, 61]. Wei et al. found that when compared patients with low PDGFRα level, those with high expression of PDGFRα showed a significantly lower OS and worse disease-free survival (DFS) with a p value of 0.005 and 0.025 respectively [60]. Moreover, PDGF-C, another ligand binding with a high affinity to PDGFRα, plays a key role in liver fibrosis through stimulating mesenchymal cell types, stellate cells included. And there has revealed a positive correlation between PDGF-C expression level and HCC staging [62] and PDGF-C except induces its own receptors, also activates intracellular signaling pathway involving PKB/Akt [63, 64]. All of these studies suggest that PDGF/PDGFR system might be a potential therapeutic target for HCC.

## IDENTIFICATION OF DRIVER MUTATIONS

The development of next-generation sequencing technology has made it become a reality to evaluate the mutated genes in HCC. In addition, frequency-based and function-based methods have been developed to distinguish driver mutations from passengers. In this section, there will be a brief introduction to the identification of driver genes in HCC.

### Frequency-based approach to identify drivers

Plentiful statistical methods have been developed to distinguish drivers from passengers. The frequency-based approach is to identify driver genes with a significantly higher mutation rates than the background mutated rates [65]. And furthermore, assessments of the mutation frequency and the distribution of nonsense, missense, and frame shift and spice sites mutations can have an indication of being an oncogene or a tumor suppressor gene, which are generally classified by the “20/20 rule”. If a gene contains mutations that more than 20% of them are missense and located recurrent positions, it can be classified as an oncogene. In contrast, a gene containing more than 20% inactivating mutations is considered to be a tumor suppressor gene.

In a study by Cleary et al. [46], the authors performed a whole-exome sequencing on 87 HCC samples and then conducted a binomial test to detect genes harboring a greater number of mutations than expected based on the background mutated rates. The authors discovered 13 new driver genes which were significantly mutated with a false discovery rate (FDR) cutoff of 5%, including the well- known oncogene, CTNNB1 and tumor suppressor, TP53, and other genes IGSF3, and ATAD3B and so on. In another study by Hirotsu et al. [66], the authors performed an ultra-deep sequencing analysis using nine pairs of samples and whole-exome sequencing using one pair of specimens. They found that missense mutations were observed in CTNNB1 (G34R, H36P) and TP53 (R110P, R273C and R283P), which were predicted to have harmful impact on protein function. All the mutations in TP53 gene are located at DNA binding domain. The results by Hirotsu et al. implied that the aberration of TP53/cell cycle and WNT/β-catenin pathways helped contribute to hepatocarcinogenesis. In addition, Nault [19] et al. illustrated that TERT promoter mutations were the most frequent somatic alterations in HCCs. And its association with CTNNB1 mutation often contributes to the malignant transformation.

However, three reasons make it difficult to accurately estimat the background mutation rates, including the variability in tumor types and sample individuals, and the characteristic of each gene. Thus, even if the right background mutated rates are obtained, false positive evaluation may exist due to the underestimating of variability.

Three shortcomings were found in the frequency-based approach. First, previous methods ignore that different kinds of mutations have a different impact on protein functions. The different specimens possess a different background mutated rates, which is the second disadvantage of previous approaches. And the last one is that the probability of silent or non-silent mutation for each base pair is variable. All the defects propel investigators to develop new method to predict driver genes more accurately. In this article, Ahrim et al. [23] developed a new method to identify carcinoma driver genes with a hypothesis that each sample has a different mutation rate and different mutation types possess separated mutation rates. The method based on this background mutation model provided a higher accuracy than previous methods.

### Function-based approach to identify drivers

The method based on frequency is not suitable for identifying driver genes mutated infrequently, yet these genes may have a vital effect on HCC. The major principle of identifying infrequent mutated driver genes is based on the functional impact on their corresponding proteins. And the assessment of functional impact is based on evolutionary conservation, changes of its secondary and tertiary structure, and biochemical similarity of the amino acid before and after mutation. The function-based approach is suitable for single sample data and studies of heterogeneity. However, there is a fact that not all aberrations in highly-conserved regions are drivers. In a research by Gnad et al. [67], the highest accuracy of previous studies based on functional impact is no more than 81%.

Recently, a new advance about functional impact identification is the combination of multi-omics data, including transcriptome and epigenome. Guichard et al. [3] performed a high-resolution copy-number and functional analysis in 125 HCC samples, finding four novel driver genes(ARID1A, RPS6KA3,NFE2L2 and IRF2) not previously reported in HCC and the inactivation of IRF2 could lead to a damaged TP53 function. In another study by Kwon et al. [68], an integrative analysis of copy number aberrations and transcriptional dysregulation data provides a significant advantage to depict the heterogeneous HCC development. And further study by Jiang et al. [69] suggested the utilization of protein-protein interaction networks was profitable to identify driver mutations. They assumed that interacting proteins generally possess the same or similar functions and finally found 33 genes related to HCC tumorigenesis and development. HCC network (HCCNet) [45, 70], an online graphic platform, integrates numerous data of proteins, mRNA and genes that related to HCC, which provides a better approach to understand the pathology and mechanism of HCC. And in a study by He et al. [71], the results showed that CTNNA3 is a tumor suppressor and can inhibit proliferation and migration of HCC cells. All these studies based on function impact implied that an integrated and comprehensive analysis of multiple-omics data was a potential method to identify driver genes in HCC.

The function-based approach provides a more accurate identification for those infrequent genes, yet it is unsatisfactory for the evaluating of HCC tumor cells growth advantage provided by mutated proteins.

## TARGETED THERAPIES FOR HCC

**Figure 1:**
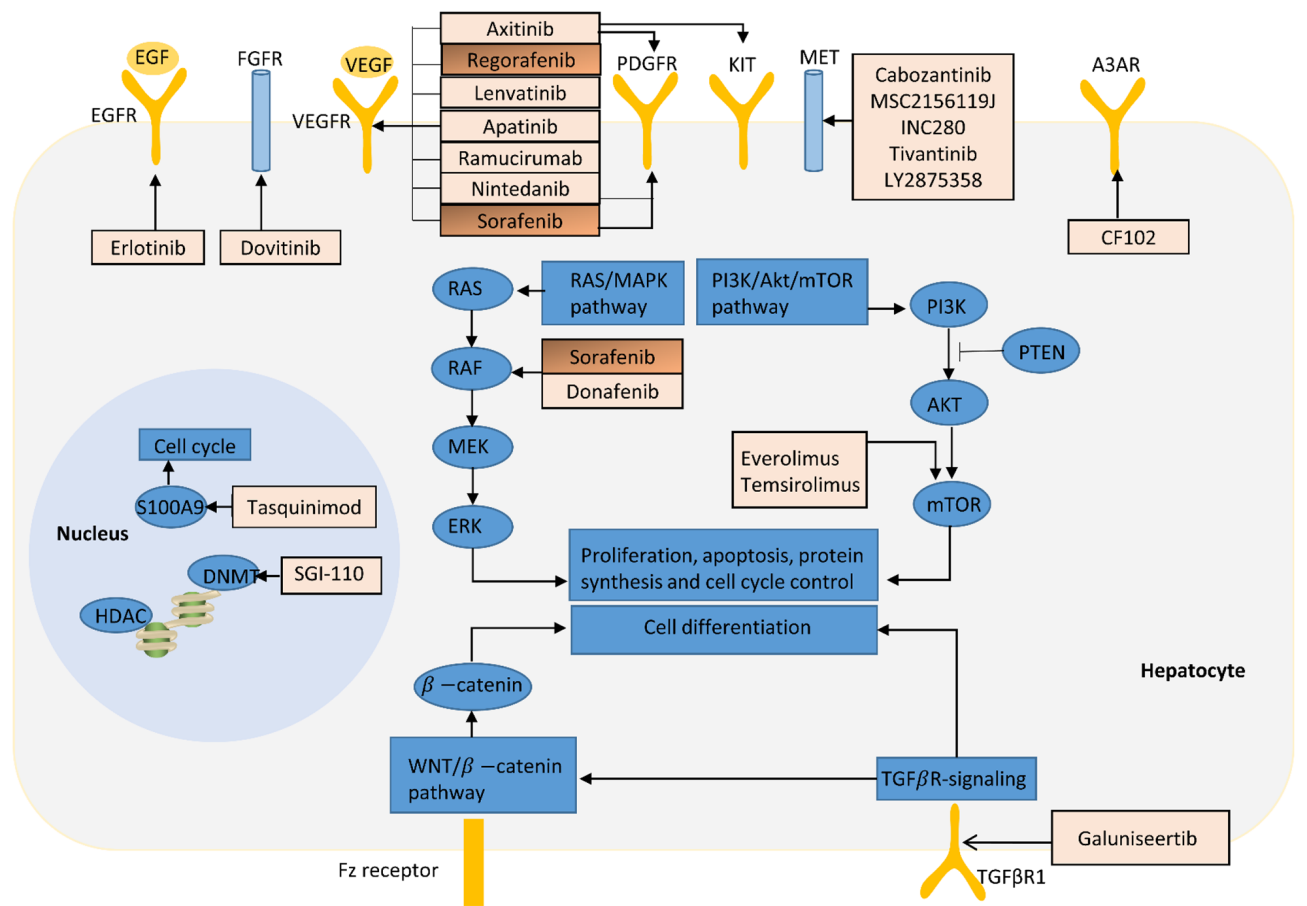
Targeted therapies for HCC and their target signaling pathways Drugs in orange boxes (sorafenib and regorafenib) have been approved by FDA for the treatment of patients in HCC, while others are being evaluated in Phase II or III clinic trials.

HCC is rather harder to find and diagnose at an early state due to the complex pathology, which makes patients lose the opportunity to avoid the malignant transformation. Besides, as for advanced HCCs, only sorafenib shows improved overall survival for approximately three months before 2017. And the development of next-sequencing technology has found numerous driver genes and affected pathways in HCC, providing a chance to develop anti-cancer management through targeted therapies [72]. Most agents are currently being tested, including anti-angiogenic agents, MET inhibitors, mTOR inhibitors and immune modulators [11]. Targeted therapies for HCC and their target signaling pathways are illustrated in Figure 1.

### Anti-angiogenic agents

As mentioned above, VEGF/VEGFR and PDGF/PDGFR are key components in tumor angiogenesis and the approval of sorafenib has opened a new epoch of anti-angiogenic therapy for HCCs.

**Sorafenib**, a small molecular inhibitor that plays a pivotal role in not only inhibiting of tumor cell proliferation and angiogenesis, but also improving the tumor cell apoptosis, has received as “Fast Track” designation by the FDA for the treatment of advanced HCC. VEGFRs, PDGFRs and Raf are all its targets. Llovet et al. [14] found that sorafenib showed a significant longer survival advantage than treatment with placebo, with an improved median OS from 7.9 to 10.7 months (hazard ratio (HR),0.69; 95% confidence interval [CI], 0.55 to 0.87; P<0.001). The median time to progression (TTP) was also significantly longer when treated with sorafenib (5.5 versus 2.8 months, p<0.001) and the disease control rates (DCR) could achieve at 43% (Table 2). And further subgroup analyses showed that sorafenib consistently improved median OS and DCR compared with placebo for patients with advanced HCC, irrespective of disease etiology, baseline tumor burden, performance status, tumor stage, and prior therapy, although they compared the sorafenib and placebo subgroups descriptively without formal statistical test [73]. Particularly, HCV-infected patients treated with sorafenib had a superior median OS (14.0 vs. 7.4 months), and among HBV-positive patients, those treated with sorafenib had a longer median OS (9.7 vs. 6.1 months), which suggested that sorafenib could improve median OS due to either etiology and patients with HCV-related HCC could derive more clinical benefit from sorafenib treatment than do patients with HBV-related HCC [73]. These findings have been subsequently replicated in a phase III randomized, double-blind, placebo-controlled trial for advanced HCC patients in Asia-Pacific region [74, 75].

**Table 2:** Summary of clinical trials of targeted therapies for advanced HCC

Treatment	Targets	Phase	Evaluable patients number	Disease control rate	Median TTP(or PFS) (months)	Median OS (months)	Reference
**Sorafenib** 400 mg bid Placebo	VEGFR, PDGFR	III	299 303	43% 32%	5.5 2.8	10.7 7.9	[14]
**Sorafenib** 400 mg bid Placebo	VEGFR, PDGFR	III	150 76	35.3% 15.8%	2.8 1.4	6.5 4.2	[74] (Asian-pacific region)
**Ramucirumab** 8 mg/kg every two weeks	VEGFR2	II	52	69.0%	4.2	12.0	[82]
**Ramucirumab** 8 mg/kg every two weeks Placebo	VEGFR2	III	45 48	67% 46%	4.1 1.7	12.9 8.0	[83] (Japan)
**Regorafenib** 160 mg once daily	VEGFR1-3, TIE2	II	36	72%	4.3	13.8	[87]
**Regorafenib** 160 mg once daily Placebo	VEGFR1-3, TIE2	III	379 194	65% 36%	3.1 1.5	10.6 7.8	[86]
**Lenvatinib** 12 mg once daily	VEGFR1-3, PDGFRβ	II	46	78%	7.4	18.7	[90]
**Tivantinib** 240 mg twice daily placebo	MET	II	71 36	44% 31%	1.6 1.4	6.6 6.2	[98]
**Tremelimumab**15 mg/kg every 90 days	CTLA4	II	31	76.4%	6.48	8.2	[114]

Abbreviations: CTLA4, cytotoxic T lymphocyte protein 4; MET, hepatocyte growth factor receptor; OS, overall survival; PDGFR: platelet-derived growth factor receptor; PFS, progression-free survival; TIE2, angiopoietin-1 receptor; TTP, time to progression.

However, further studies showed that management with sorafenib was frequently accompanied with some adverse events, such as fatigue (3.4%), diarrhea (8%), hand–foot skin reaction (8%) and abdominal pain (2%), thus toxicity monitoring should be performed every 4-6 weeks for patients treated with sorafenib [74, 76]. Moreover, sorafenib will be limited for patients with poor liver function (Child-Pugh>B7) and severe cardiovascular disease, and the 60 days of median using duration only improved median OS with nearly 3 months, though the cost of dose-adjusted sorafenib treatment, €34,534 per quality-adjusted life year (QALY) is lower than the generally accepted threshold €46000 per QALY [77–79]. Thus, further studies are needed to evaluate sorafenib in combination with other targeted therapies.

**Ramucirumab**, a recombinant human IgG1 monoclonal antibody, has a high binding affinity to VEGFR2, which prevents the specific binding between VEGF and VEGFR2 to inhibit angiogenesis. This agent was approved by FDA for treatment of advanced gastric or gastro-esophageal junction adenocarcinoma and metastatic non-small-cell lung carcinoma in 2014. And in recent years, some studies have found that ramucirumab showed an antitumor activity in advanced HCCs and the adverse events were acceptable and manageable, fatigue (22.8%), diarrhea (21.1%), hypertension (17.1%) and leukopenia (14%) [80, 81]. A phase II and biomarker Study for forty-two patients showed that first-line treatment with ramucirumab showed a median OS and PFS of 12.0 and 4.0 months respectively [82]. Although the observed PFS was shorter than 5-month target, that the overall response rate of 9.5% and the OS of 12 months indicated potential antitumor activity of ramucirumab in advanced HCC [82].

When ramucirumab in second-line treatment, the results of medial OS were inconsistent. Zhu et al. [57] found that median OS in ramucirumab group showed no significance when treated with placebo (p=0.14), while in another study the significant improvement in median OS was 4.9 months (p=0.0416) [83], although the evaluation results of PFS was both significant improved. And Zhu's study demonstrated that ramucirumab treatment possibly showed a greater OS benefit in advanced patients with HBV than HCV (HR: 0·79 vs.0·88), which was validated in subsequent Japanese subgroup analysis by Kudo’ group [57, 83]. Ramucirumab proved also effective to significantly improve approximately 4 months median OS (p<0.05) for patients with an alpha-fetoprotein ≥400 ng/mL, whose production was increased in HCCs, chronic hepatitis and cirrhosis [84]. After treatment with ramucirumab, the serum VEGF showed an increase tendency due to the blockade of VEGFR2, which suggested that VEGF might be a biomarker in anti-angiogenic therapy [82].

**Regorafenib**, a new oral multikinase inhibitor that targets VEGFR1-3, PDGFR β, FGFR and tyrosine kinase with immunoglobulin and epidermal growth factor homology domain 2 (TIE2, also known as angiopoietin-1 receptor), showed a broad spectrum of antitumor activity (inhibiting tumor angiogenesis and cell proliferation) as second-line treatment for intermediate or advanced HCC (stage B or C) [85]. Although sorafenib could improve median OS from 7.9 to 10.7 months, it was unfortunate that disease progressed when treated with sorafenib. Bruix et al. [86] assessed the efficacy and safety of regorafenib treatment for HCCs whose disease still progressed following the first-line sorafenib treatment. In this phase III trial, median OS was 10.6 months 95% (95% CI, 9·1–12·1) for regorafenib versus 7.8 months (95% CI, 6.3-8.8) for placebo and regorafenib improved OS with a HR of 0.63 (95% CI, 0·50–0·79; one-sided p<0·0001). Moreover, this survival benefit of regorafenib was maintained in subgroup analysis (aetiology included) compared with placebo, as reflected by HR of 0.58 and 0.79 for HBV- and HCV- positive patients respectively. The significant improvement in PFS, TTP, and objective response, and DCR were also derived, although 3.6 months duration time was needed [86, 87]. The adverse events of regorafenib were quite similar to sorafenib, including hypertension (15%), hand-foot skin reaction (13%), and fatigue (9%). Based on these findings, FDA expanded the indications of regorafenib to include the treatment of patients with HCC in April 2017.

**Lenvatinib**, a multi-targeted inhibitor of VEGFR1-3, PDGFR α and FGFR1-4, has been approved by FDA for the treatment of patients with locally recurrent or metastatic, progressive, radioactive iodine-refractory differentiated thyroid cancer in 2015. And in recent years, several clinical trials were performed to assess the antitumor activity and safety of lenvatinib in patients with advanced HCC [88, 89]. Ikeda et al. [90] designed a phase II study to evaluate this agent in advanced HCCs and found that median OS was 18.7 months (95 % CI: 12.7–25.1) and DCR was 78%, which might be prior to sorafenib. However, there was a drawback that this was a single-arm design study, thus the efficacy and safety results might be influenced by selection of patients. 12-mg once daily is recommended dose with an acceptable toxicity and some manageable adverse events, but early dose modification was necessary in patients with lower body weight [90, 91]. A multicenter, open-label, phase 3 trial (NCT01761266) is ongoing to compare the efficacy and safety of lenvatinib versus sorafenib in first-line treatment of patients with advanced HCC.

**Apatinib** and **axitinib** (both targeting VEGFR) are currently being investigated in antiangiogenic therapies for advanced HCCs. Apatinib has been proved effective in many solid tumors, such as gastric cancer, breast cancer and non-small-cell lung cancer [92]. Apatinib might also be an optional agent for HCCs at stage C because the median PFS was more than 8 months and the alpha-fetoprotein level was significantly decreased [93]. A single-arm phase II trial of second-line axitinib demonstrated that this inhibitor could achieve 42.3% of tumor control at 16 weeks and further study should be performed to combine the biomarker responses [94].

### MET inhibitors

MET, the receptor for HGF, can be activated to promote migration and invasion of tumor cells and provides a potential target for treating HCC. Although the overexpression of MET is rare (1-4%), it provides an underlying target for HCC treatment [25, 95] There are two small molecular inhibitors being evaluated in HCC treatment, cabozantinib and tivantinib.

**Tivantinib**, also known as ARQ197, was defined as a selective, non-ATP competitive MET inhibitor with a broad spectrum of antitumor activity and was currently being tested for the treatment of advanced HCC with the available data of phase I and II [96]. The first study of tivantinib in patients with advanced HCC was a phase IB trial conducted by Santoro et al [97]. In this trial, median treatment duration was 1.8 months and stable disease was in 9 of 16 evaluable patients (56%) with some drug-related adverse events, such as neutropenia, anemia, leucopenia, asthenia, anorexia, diarrhea, and fatigue. Based on positive phase I results, a randomized, placebo-controlled phase II study was conducted [98]. When treated with tivantinib, there was a significant improvement in median TTP from 1.4 to 1.6 months (p=0.04) and the overall disease control rate at 12 weeks was 68%. And the median duration time was 1.8 months (range, 1.6-5.3 months), slightly shorter than sorafenib. Interestingly, median OS was 7.2 months for patients with high-MET treated with tivantinib versus 3.8 months treated with placebo (HR 0.38, 95% CI 0.18–0.81; p=0.01) while there were no not significant differences among patients with low-MET treated with tivantinib and placebo respectively (HR 0.96, 95% CI 0.43–2.14; p=0.92) [98]. Thus, further study should focus on high-MET patients. Based on these findings, two randomized double-blind placebo-controlled phase III trial (NCT01755767 and NCT02029157) are ongoing to evaluate tivantinib in patients with MET-high advanced HCC, with the primary endpoints of OS and PFS, respectively [99].

**Cabozantinib**, a receptor tyrosine kinase inhibitor that targets VEGFR and MET, approved by FDA for treating the patients with metastatic medullary thyroid cancer in 2012 and advanced renal cell carcinoma in 2016 respectively. This agent has achieved a significant improvement of median PFS for 7.2 months (hazard ratio, 0.28; 95% CI, 0.19 to 0.40; P<0 .001) for patients with medullary thyroid cancer [100]. The antitumor activity of cabozantinib was also investigated in HCCs, which inhibits the migration and invasion of tumor cells by blocking the HGF- mediated MET signaling [58]. A phase II trial of cabozantinib in 41 HCC patients showed that objective response rate at 12 weeks was 5% and the DCR was 66% [101]. In this trial, the antitumor activity of cabozantinib in advanced HCC was further supported by the 35% patients achieving > 50% alpha-fetoprotein reduction. Based on these preliminary signs of clinical activity, a phase III randomized double-blind controlled trial (NCT01908426) has been initiated to compare cabozantinib to placebo in patients with advanced HCC who have received prior sorafenib therapy.

### m-TOR inhibitors

**Everolimus** is an m-TOR inhibitor that reduces the activity of effectors downstream, which subsequently induces cell growth arrest and apoptosis. And it has been approved by FDA for the treatment of postmenopausal women with advanced hormone receptor-positive, HER2-negative breast cancer in combination with exemestane; adults with metastatic renal cell carcinoma that progressed on previous VEGF-targeted therapy; and pediatric and adult patients with unresectable subependymal giant cell astrocytoma [102]. In recent years, the antitumor activity of everolimus is being evaluated in patients with advanced HCC [103, 104]. A phase I/II study was conducted to investigate the safety, efficacy, pharmacokinetics, pharmacogenetics and feasibility of everolimus in advanced HCC patients [105, 106]. In Shiah's study, the daily and weekly maximum tolerated doses of everolimus in patients with advanced HCC was 7.5 mg and 70 mg respectively [106]. And at these doses, everolimus showed acceptable tolerability and preliminary evidence of clinical antitumor activity. As for response and survival, patients in the daily schedule had a higher DCR, longer median PFS and OS than patients in weekly schedule (71.4 vs. 67.4%; 16.0 vs. 8.3 weeks; 33.4 vs. 24.6 weeks respectively), which demonstrated that 7.5 mg daily was recommended for future studies [106]. However, in a phase III clinical trial, there was no difference in the OS between everolimus and placebo group (7.6 vs. 7.3 months; HR, 1.05; 95% CI, 0.86-1.27; P=0.68) and 91.4 patients of death was due to disease progression, which showed that everolimus did not improve OS in adult patients with advanced HCC after failure of sorafenib treatment [107, 108].

**Temsirolimus** is an intravenous drug for the treatment of renal cell carcinoma and approved by the FDA in late May 2007 [109]. Temsirolimus binds to an intracellular protein (FKBP-12), and the protein-drug complex inhibits the activity of mTOR that controls cell division [110]. Yeo et al. conducted a study to determine dose limiting toxicity and maximum tolerated dose of temsirolimus in HCC patients in phase I and to assess antitumor activity of temsirolimus in phase II [111]. In this trial, the maximum tolerated dose was 25 mg weekly, which is the approved dose for metastatic renal cell carcinoma. And amongst the 35 assessable patients, there were 1 partial response and 20 stable disease and the median OS was 8.89 months (95% CI, 5.89-13.30) [111]. Moreover, further study showed that the combination of temsirolimus and sorafenib had an acceptable safety profile in patients with advanced HCC, but that whether there was a benefit from the combination of mTOR inhibition with sorafenib over single agent didn't be illustrated [112].

### Immune-modulators

**Tremelimumab**, a monoclonal antibody, binds to cytotoxic T lymphocyte protein 4 (CTLA4) to stimulate human immune system and has been undergoing human trials for the treatment of various cancers but has not attained approval for any [113]. Sangro et al. [114] performed a phase II clinical trial to evaluate the efficacy and safety profile of tremelimumab treated in patients with advanced HCC and chronic HCV infection. In their study, median TTP was 6.48 months and median OS was 8.2 months. And moreover, the HCV viral load was significantly dropped, which indicated that tremelimumab not only has an antitumor activity but also antiviral effects. The combination treatment of tremelimumab and ablation for advanced HCC can achieve a median 7.4 months of TTP and 12.3 months of OS respectively, which was superior to single tremelimumab treatment [113]. Ipilimumab and nivolumab were also investigated to assess the efficacy and safety for advanced HCCs [115, 116].

Targeted therapies mentioned above, only two drugs (sorafenib and regorafenib) have been approved by FDA for the treatment of HCC in November 2007 and April 2017 respectively, while other agents, such as cabozantinib, everolimus, ramucirumab and temsirolimus, are still on clinical trials.

## CHALLENGES

HCC is a kind of complex disease that has multiple risk factors, including hepatitis B, hepatitis C, alcohol intake and obesity. To date, it is much known about etiologic agents, yet the molecular mechanism of HCC is still negligible. The development of next-sequencing technology has revolutionized the reveal of genetic aberrant of HCC.

The intra-tumor heterogeneity of HCC is a major element making it difficult to propose a standardized management strategy for HCC. Thus, the future therapy direction of HCC will be focused on personalized medicine and it is essential to exactly identify driver genes and pathways in different HCC patients. The two crucial approaches for recognizing driver genes are based on mutation frequency and functional impact on its encoding proteins. The frequency-based methods can directly suggest the selective growth advantage is conferred [117]. In contrast, the function methods can identify driver mutations from single-sample data and are practicable for both rarely and frequently mutated genes. However, the latter methods can reduce the false positive comparing to the frequency-based approaches [118]. Further studies demonstrate that the integrated molecular network analysis provides a potential insight to elucidate underlying genetic mechanism of HCC. The interacting proteins share same or similar functions and may be involved in same pathway according “guilt by association” rule. Jiang et al. [69] identified 33 HCC related genes based on protein-protein interaction networks and significantly enriched several pathways, MAPK signaling, cell cycle, and host immune responses included. Some novel genes such as CDT1, GRPEL1 and RRM2B were first identified in these pathways, which may be novel HCC candidate genes. All these findings suggest molecular network provides a new insight to identify driver genes in HCC.

Although numerous drivers have been identified in HCC, most of these drivers have not been translated into effective treatment to date, such as TERT, TP 53 and CTNNB1. Thus, the discovery of agents targeting these mutations represents a significant breakthrough for HCC treatment [16]. Many drugs are currently being evaluated for HCC, yet no one could achieve more 18.7 months OS than lenvatinib [90]. So, it is urgent to develop new treatment strategies for HCC. In addition, HCC is a complex disease with multiple pathological bases, which implies that the combination of multiple drugs or multi-targets drugs may be a future direction for HCC treatment [72, 119]. The research and development of new drugs is a long process with high input, high risk and uncertain profile [120]. Thus, combination therapeutics might be a better prospect and has been investigated in several complex diseases, such as melanoma, colorectal cancer, pancreatic cancer and breast cancer [121–123]. Median OS was 25.1 months in dabrafenib and trametinib group for melanoma versus 18.7 months (p=0.0107) in the dabrafenib only group [122]. The improved outcomes (better efficacy, less toxicity and less drug resistance) indicated the combination of multiple drugs might also be applicable for HCC.

## Abbreviations

ARID1A: AT-rich interactive domain-containing protein 1A
ARID2: AT-rich interactive domain 2
CCND1: cyclin D1
CDKN2A: cyclin-dependent kinase inhibitor 2A
CTLA4: cytotoxic T lymphocyte protein 4
CTNNB1: β-catenin
DCR: disease control rates
DFS: disease-free survival
FGF19: fibroblast growth factor 19
HCC: hepatocellular carcinoma
HGF: hepatocyte growth factor
IGFR: insulin-like growth factor receptor
KEAP1: kelch-like ECH-associated protein 1
NFE2L2: nuclear factor erythroid 2-related factor 2
OS: overall survival
PDGF: platelet-derived growth factor
PFS: progression-free survival
PTEN: phosphatase and tensin homologue
TACE: transarterial chemoembolization
TERT: telomerase reverse transcriptase
TP53: tumor protein 53
TTP: time to progression
VEGFA: vascular endothelial growth factor A
